# Cells from discarded dressings differentiate chronic from acute wounds in patients with Epidermolysis Bullosa

**DOI:** 10.1038/s41598-020-71794-1

**Published:** 2020-09-15

**Authors:** Ignacia Fuentes, Christina Guttmann-Gruber, Birgit Tockner, Anja Diem, Alfred Klausegger, Glenda Cofré-Araneda, Olga Figuera, Yessia Hidalgo, Pilar Morandé, Francis Palisson, Boris Rebolledo-Jaramillo, María Joao Yubero, Raymond J. Cho, Heather I. Rishel, M. Peter Marinkovich, Joyce M. C. Teng, Timothy G. Webster, Marco Prisco, Luis H. Eraso, Josefina Piñon Hofbauer, Andrew P. South

**Affiliations:** 1 DEBRA Chile, Francisco de Villagra 392, Ñuñoa, Santiago, Chile; 2 Centro de Genética Y Genómica, Facultad de Medicina Clínica Alemana, Universidad de Desarrollo, Santiago, Chile; 3 EB House Austria, Research Program for Molecular Therapy of Genodermatoses, Department of Dermatology and Allergology, University Hospital of the Paracelsus Medical University, Salzburg, Austria; 4 EB House Austria, Outpatient Unit, Department of Dermatology and Allergology, University Hospital of the Paracelsus Medical University, Salzburg, Austria; 5 Consorcio Regenero, Chilean Consortium for Regenerative Medicine, 7620157 Santiago, Chile; 6 Cells for Cells, 7620157 Santiago, Chile; 7 Faculty of Medicine, Universidad de Los Andes, 7620001 Santiago, Las Condes Chile; 8 Facultad de Medicina Clínica Alemana, Universidad de Desarrollo, Santiago, Chile; 9 UCSF Dermatology, San Francisco, CA USA; 10 Dermatology Department, Stanford University School of Medicine, Stanford, CA USA; 11 Dermatology Service, VA Medical Center, Palo Alto, CA USA; 12 Dermatology and Cutaneous Biology, Thomas Jefferson University, Bluemle Life Sciences Building, Room 406, 233 South Tenth Street, Philadelphia, PA 19107 USA; 13 Vascular Medicine, Thomas Jefferson University, Philadelphia, PA USA; 14 Joel and Joan Center for Fibrotic Diseases Research, Thomas Jefferson University, Philadelphia, PA USA; 15 Sydney Kimmel Cancer Center, Thomas Jefferson University, Philadelphia, PA USA

**Keywords:** Cell biology, Immunology, Molecular biology, Diseases

## Abstract

Impaired wound healing complicates a wide range of diseases and represents a major cost to healthcare systems. Here we describe the use of discarded wound dressings as a novel, cost effective, accessible, and non-invasive method of isolating viable human cells present at the site of skin wounds. By analyzing 133 discarded wound dressings from 51 patients with the inherited skin-blistering disease epidermolysis bullosa (EB), we show that large numbers of cells, often in excess of 100 million per day, continually infiltrate wound dressings. We show, that the method is able to differentiate chronic from acute wounds, identifying significant increases in granulocytes in chronic wounds, and we show that patients with the junctional form of EB have significantly more cells infiltrating their wounds compared with patients with recessive dystrophic EB. Finally, we identify subsets of granulocytes and T lymphocytes present in all wounds paving the way for single cell profiling of innate and adaptive immune cells with relevance to wound pathologies. In summary, our study delineates findings in EB that have potential relevance for all chronic wounds, and presents a method of cellular isolation that has wide reaching clinical application.

## Introduction

Chronic wounds, often characterized as wounds that remain open for three months or more, cost an estimated $10–20 billion dollars per year for the US healthcare system alone^1^. Globally, the financial burden of chronic wounds is increasing due to the increasing age of populations, and the increasing incidence of obesity, diabetes and peripheral arterial disease^2–4^. From a patient perspective non-healing wounds cause significant morbidity and mortality, the burden of which has been compared to cancer^5^.

Wound management represents a significant challenge to health care professionals through lack of efficient markers that predict chronicity and direct appropriate therapy, which is often limited to debridement, a wide range of dressings in conjunction with topical agents, and the use of negative pressure^6^.

Chronic wounds result from disruption to one of four orchestrated phases that normal, acute wound healing undergoes: hemostasis, inflammation, proliferation, and remodeling^7^. Dysregulation of any of these steps leads to non-healing ulcers or excessive scarring. Although considerable efforts are moving our understanding of the biology of wound healing forward, the exact mechanisms driving chronicity beyond an inability to resolve inflammation remains unclear and represents a barrier to therapeutic development. Prior analysis of chronic wounds in patients have focused on profiling wound exudates, or blister fluids^8^, sometimes isolated from discarded wound dressings^9,10^. Many studies have identified evidence of increased cytokines associated with neutrophils in chronic wounds, suggesting that persistence of neutrophils, one of the first immune cell populations to migrate into wounds during the inflammatory stage, is a feature of chronic wounds^11–13^. These observations in patients are supported, and often directed, by studies in rodents^12,14^. Murine studies have shown that lack of macrophages^14,15^, rather than lack of neutrophils^16^ delays wound healing and suggests that the inability of macrophages to clear neutrophils present in a wound is a limiting factor for normal resolution of inflammation and subsequent healing. Data from genetically engineered mouse models also implicates so called “invariant NKT cells” in clearance of neutrophils and progression of wound healing^17^.

Epidermolysis bullosa (EB) is a group of inherited skin diseases where wound healing is of primary clinical importance due to mutations in genes that encode proteins important for maintaining the integrity and structure of the skin and mucosa^18^. Two severe forms of the disease are recessive dystrophic EB (RDEB) and junctional EB (JEB). Subsequent skin fragility in EB patients generates extensive blisters and erosions, and the resulting wounds in EB individuals are usually slow to heal or frequently become chronic, often for many years. For patients with RDEB, sites of chronic wounds are especially concerning as they are at high risk of developing aggressive squamous cell carcinomas early in life. Interestingly, in the context of wound healing, there is a high inter- and intra-patient variability, and certain areas of skin in an individual EB patient may experience constant wounding and healing. Ultimately, it is very difficult to predict which wound in a given patient will heal and which will not^19^. Identifying the reasons why certain wounds heal and why others do not will greatly increase our understanding of EB wound healing and will direct clinical management as well as therapeutic discovery.

To this end, we have investigated the use of > 100 discarded wound dressings from 51 patients as a non-invasive method of sampling cells present at acute and chronic wounds. We show that large numbers of cells can be isolated from discarded wound dressings (up to 6.2 × 10^8^) and that these cells can be used for genetic testing and, more importantly in the context of understanding barriers to wound resolution, can identify differences in cellular composition between acute and chronic wounds.

## Results

### Large numbers of viable cells are readily recovered from discarded wound dressings

**Figure 1:**
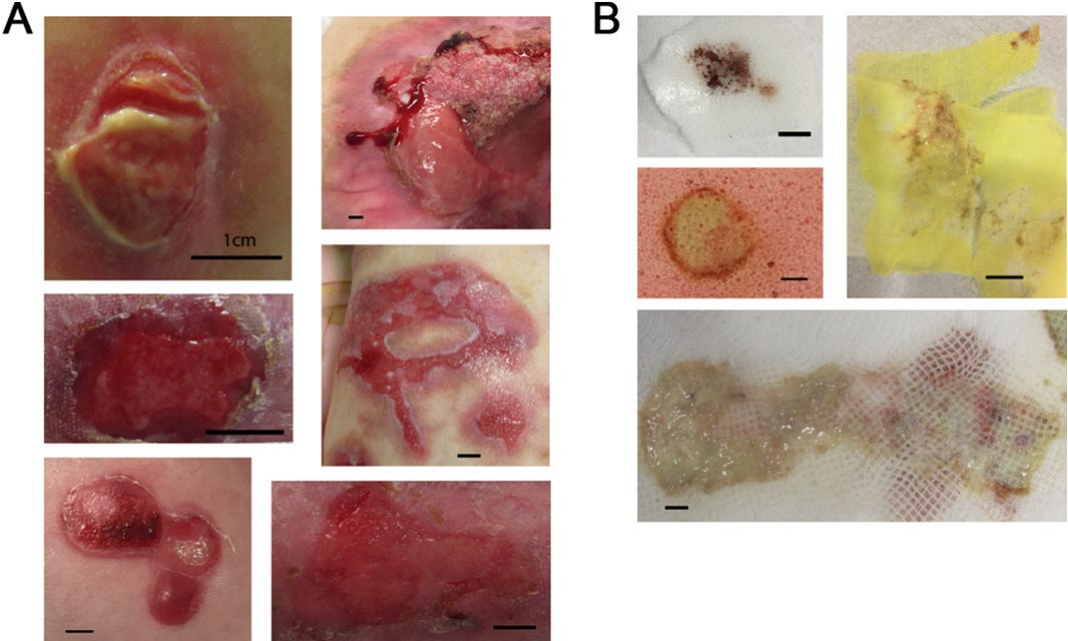
Wounds (**A**) and wound dressings (**B**). Pictures are described as epidermolysis bullosa (EB) sub type (JEB = junctional EB, RDEB = recessive dystrophic EB), dressing type, patient ID (Supplementary Table 1), wound age and total viable cells isolated where available. (**A**) Upper left: JEB, Xeroform, pEB85W1_1, > 3 months. Upper right: RDEB, Xeroform, pEB1W1_1, 2 months, 52.125 × 10^6^ cells recovered. Middle left: RDEB, Xeroform, pEB2W1_1, 2 weeks. Middle right: JEB, Polymem, pEB256W3, > 3 months, 129.8 × 10^6^ cells recovered. Bottom left: JEB, Mepilex Transfer, pEB278W1_2, 5 days, no cells isolated. Bottom right: RDEB, Mepilex Transfer, pEB110W1, 7 days, 2.375 × 10^6^ cells isolated. (**B**) Upper left: JEB, Mepilex Transfer, pEB278W1_2, 5 days, no cells isolated. Middle left: JEB, Polymem, pEB278W1_1, 14 days, 140 × 10^6^ cells recovered. Upper right: JEB, Xeroform, pEB85W1_2, > 3 months, 66.7 × 10^6^ cells recovered. Bottom: RDEB, Jelonet, pEB107W3, 2–3 weeks, 60 × 10^6^ cells recovered. Bar = 1 cm.

**Figure 2:**
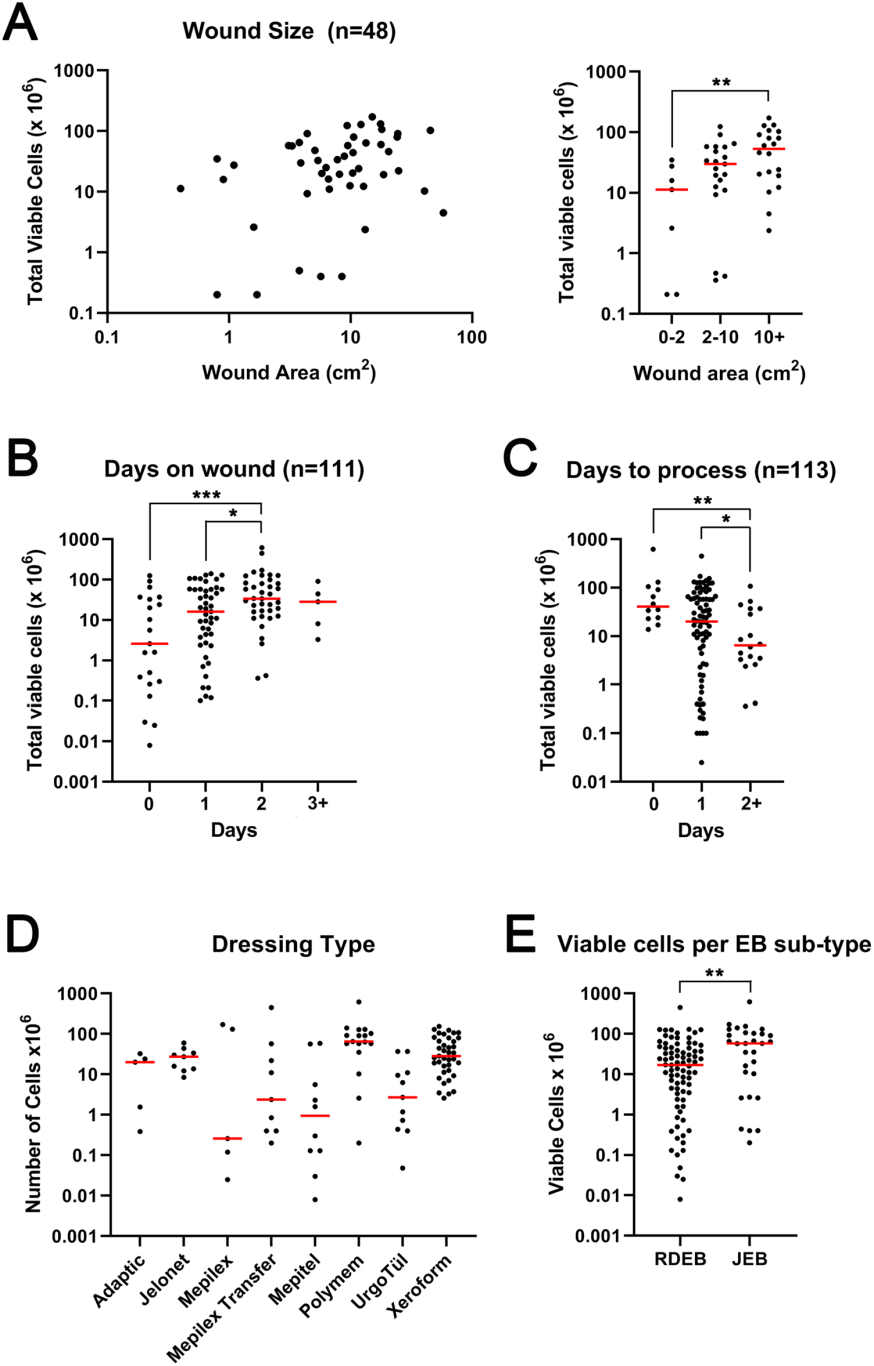
Total viable cells isolated from wound dressings is dependent on wound size, days in contact with the wound, days in storage before processing, and sub type of Epidermolysis Bullosa. (**A**) Left graph shows total viable cells recovered (y-axis) plotted against wound area (x-axis). Right graph shows the same data grouped by wound size. (**B**) Graph shows total viable cells recovered from individual dressings present on wounds for the indicated time point (x-axis). (**C**) Graph shows total viable cells recovered from individual dressings after storing for the indicated time point (x-axis). (**D**) Graph shows total viable cells recovered from individual dressings grouped by dressing type (x-axis). (**E**) Graph shows total viable cells recovered from individual dressings from either patients with junctional epidermolysis bullosa (n = 30, JEB) or recessive dystrophic epidermolysis bullosa (n = 82, RDEB). Red bar indicates median. *p < 0.05, **p < 0.01, ***p < 0.001, Mann Whitney U test. Graphs made in GraphPad Prism 8, https://www.graphpad.com/scientific-software/prism/.

To determine whether viable cells could be isolated from discarded wound dressings we collected cellular material and assessed viability using trypan blue exclusion from a total of 126 discarded wound dressings from 48 patients, see Fig. 1 for examples of wounds and dressings used for this study and Supplementary Table 1 for full details. As described in “Materials and methods” section, dressings were either processed at the host institution or shipped from clinical sites to processing laboratories. Overall, we isolated a range of 0–6.2 × 10^8^ viable cells per dressing (Supplementary Table 1) with 113 samples yielding viable cells and 13 samples recovering no viable cells (10.3%). Of note, few or no viable cells were recovered from wound dressings containing silver, such as Mepilex Ag (Mölnlycke), or wound dressings where either little wound material was evident, or where the wound material was desiccated. A correlation was identified between wound size and viable cells for 48 samples where photographs were available for accurate measurement (Spearman correlation, *p* = 0.018, r = 0.34). Smaller wounds (< 2 cm^2^) recovered significantly less cells than larger (≥ 10 cm^2^) wounds (median 11.25 × 10^6^ vs. 53 × 10^6^ cells, *p* = 0.0091) (Fig. 2A). The range of cells recovered for these 48 samples was 0.05–43.5 × 10^6^ cells per cm^2^. The longer the dressing was in contact with the wound the more viable cells were recovered (Spearman correlation, *p* = 0.0003, r = 0.34) (Fig. 2B) and the longer the dressings were stored before processing the fewer viable cells were recovered (Spearman Rank correlation, *p* = 0.017, r = − 0.22) (Fig. 2C). No significant difference was observed in dressing type, although Jelonet and Xeroform dressings recovered more consistent numbers (Fig. 2D). Interestingly JEB patient wounds (n = 30) yielded more cells than RDEB patient wounds (n = 82, median 17 × 10^6^ compared with 58 × 10^6^, *p* = 0.007) (Fig. 2E) and no difference in wound size was observed comparing JEB with RDEB. No correlation was observed between viable cell number and age of patient or age of wounds and no obvious bias with relation to time to process, time on wound, or other variables that might infleunce differences between RDEB and JEB wound cell isolations were observed (Supplementary Table 1).

### Viable adherent cells are readily isolated from wound dressings

We initiated this study with the idea of isolating viable keratinocytes from wound dressings as a potential source for identifying pre-cancerous lesions in patients with recessive dystrophic epidermolysis bullosa. Patients with this disease are at increased risk of developing squamous cell carcinoma at the site of chronic wounds and being able to identify those wounds at greatest risk of cancer would aid clinical management. To this end, however, we were not successful in isolating proliferative keratinocytes, despite multiple different approaches used (not shown). Instead, we readily isolated cells that exhibited features of squamous differentiation but which did not proliferate, even if they were adherent (a rare event) (Supplementary Fig. 1A). We were, however, successful in isolating adherent cells with a range of morphological appearances, some of which readily proliferated and expanded in culture (Supplementary Fig. 1B). Populations of cells that proliferated and expanded in culture were positive for markers consistent with a fibroblast lineage including fibroblast actívation protein alpha and alpha smooth muscle actin. Of note, proliferation of adherent cells was not observed in all isolations (19/63 attempts). Some isolations were lost to uncontrolled infections (16/63) and some did not proliferate (28/63), even after months in tissue culture. No obvious correlation with wound characteristics and our ability to culture adherent cells from dressing was apparent (Supplementary Table 1). It was evident after our first attempts at isolating adherent cells that the vast majority of viable cells present in wound dressings are non-adherent (Supplementary Fig. 1C).

### Immune cell profiling of wounds

**Figure 3:**
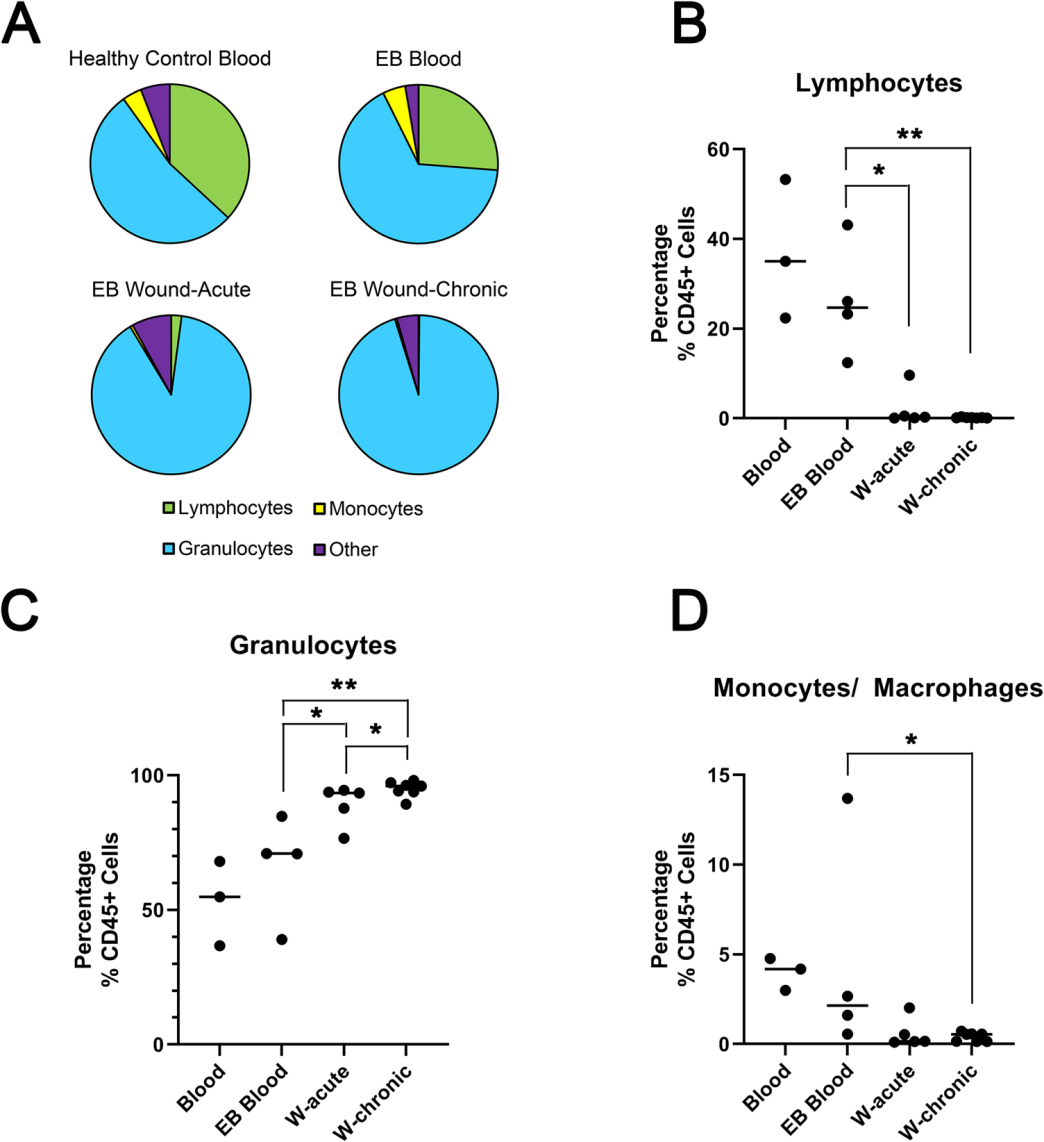
Proportion of lymphocytes, monocytes and granulocytes isolated from wound dressings. (**A**) pie charts showing distribution of cells types identified in healthy control blood (n = 3); EB blood (n = 4), acute wounds (n = 5) and chronic wounds (n = 7). (**B**) Graph shows the percentage of CD45 positive cells identified as lymphocytes. (**C**) Graph shows the percentage of CD45 positive cells identified as granulocytes. (**D**) Graph shows the percentage of CD45 positive cells identified as monocytes. Bar shows median. *p < 0.05, **p < 0.01, Mann Whitney U test. Graphs made in GraphPad Prism 8, https://www.graphpad.com/scientific-software/prism/.

We next sought to profile isolated wound cells using a panel of antibodies directed at ascertaining the immunological profile of separate lymphocyte, monocyte/macrophage, and granulocyte populations (Supplementary Table 2 and Supplementary Fig. 2A). For comparison we isolated leukocytes from peripheral blood at the time of dressing sampling in four patients and we also isolated peripheral blood leukocytes from three healthy donors. Initial profiling of wounds identified a wide range of cell viability (25–94%, Supplementary Fig. 3A and Supplementary Table 3) and cells isolated from discarded dressings had a much higher degree of variation based on the forward/side scatter compared with blood (Supplementary Fig. 2B). The proportion of cells positive for CD45 (a highly abundant protein on the cell surface of all nucleated blood cells and absent in other cell populations) in wound isolations showed a wide range (5–96%) compared with peripheral blood (93–100%), with significantly less CD45+ cells in wounds compared with peripheral blood (Supplementary Fig. 3B). There were significantly less lymphocytes in wound isolations (Fig. 3A,B) and significantly more granulocytes in chronic wounds compared with acute wounds and peripheral blood (Fig. 3A,C). There were significantly less monocytes/macrophages in chronic wounds compared with peripheral blood (Fig. 3A,D). It should be noted overall, populations tended to be less defined in wound isolates when compared with whole blood, and in particular the monocyte fraction appeared very close to the granulocyte fraction (Supplementary Fig. 2B).

**Figure 4:**
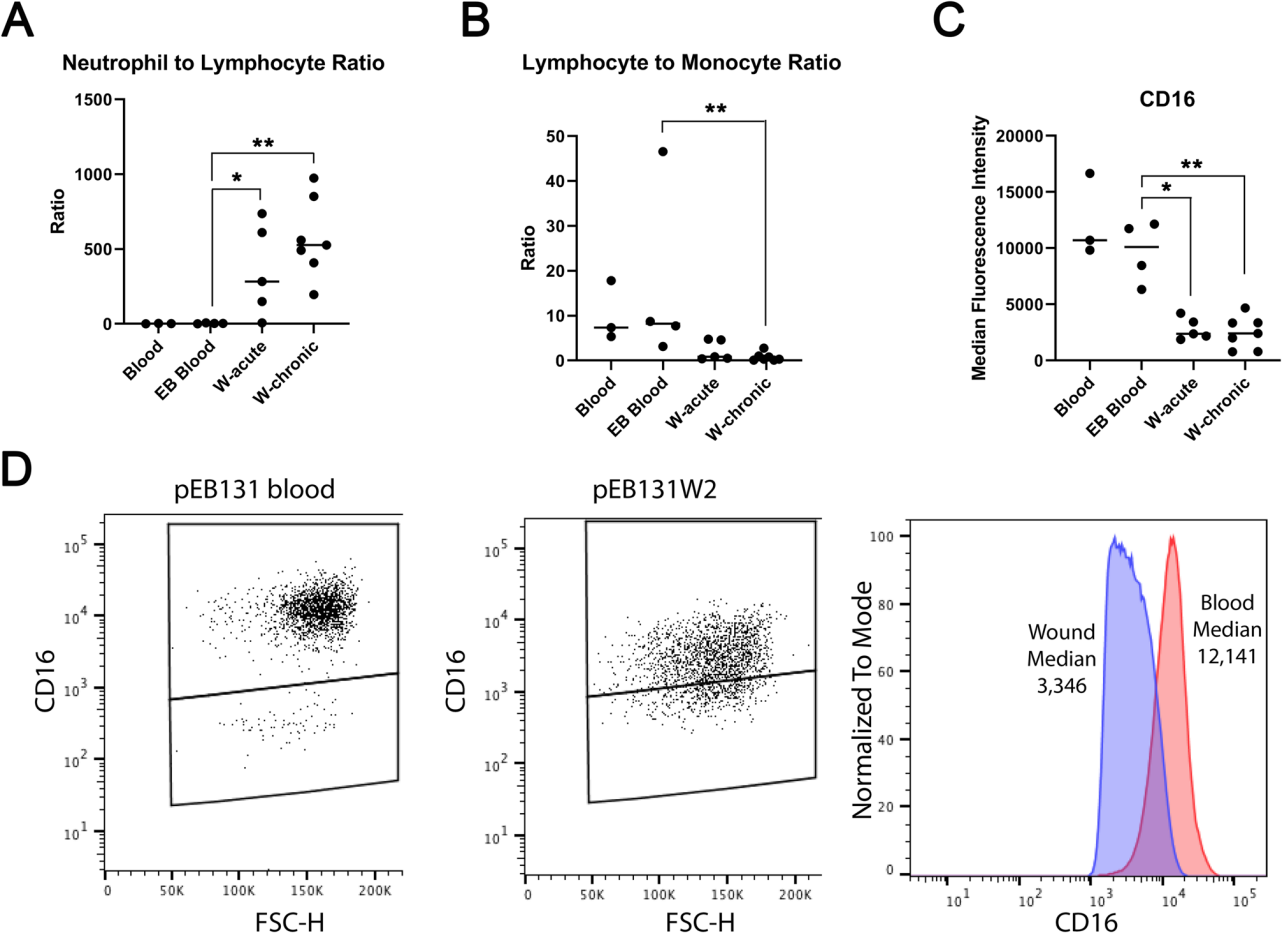
Neutrophil-to-lymphocyte and lymphocyte-to-monocyte ratios, as well as CD16 median fluorescent intensity in wound dressing isolates. (**A**) Graph shows ratio of neutrophils to lymphocytes identified in healthy control blood (Blood, n = 3); EB blood (n = 4), acute wounds (W-acute, n = 5) and chronic wounds (W-chronic, n = 7). (**B**) Graph shows ratio of lymphocytes to monocytes identified in healthy control blood, EB blood, and acute and chronic wounds. (**C**) Graph shows median CD16 fluorescent intensity identified in CD16 positive granulocytes from healthy control blood, EB blood and acute and chronic wounds. Bar indicates median. *p < 0.05, **p < 0.01, Mann Whitney U test. (**D**) Representative example showing the fluorescence shift of CD16 granulocytes expression in blood (left plot), in an EB wound (middle plot) and the median of both positive events plotted together as a histogram (right). Graphs made in GraphPad Prism 8, https://www.graphpad.com/scientific-software/prism/.

The neutrophil-to-lymphocyte ratio (CD45+/CD16+ granulocytes: CD45+/CD3+ lymphocytes), previously used as a surrogate marker of systemic inflammation and recently shown to be increased in the circulation of patients with chronic diabetic foot ulcers^20^, was significantly greater in wound isolates but was not significantly different between acute and chronic wounds, or between EB peripheral blood and healthy control peripheral blood (Fig. 4A). Similarly, the lymphocyte to monocyte ratio was significantly lower in wounds compared with peripheral blood but was not significantly different between chronic and acute wounds (Fig. 4B). Of note the overall fluorescent intensity of CD16 positive cells (of the granulocyte population), previously associated with subsets of neutrophils^21^, was significantly lower in wound isolates compared with peripheral blood (Fig. 4C,D).

**Figure 5:**
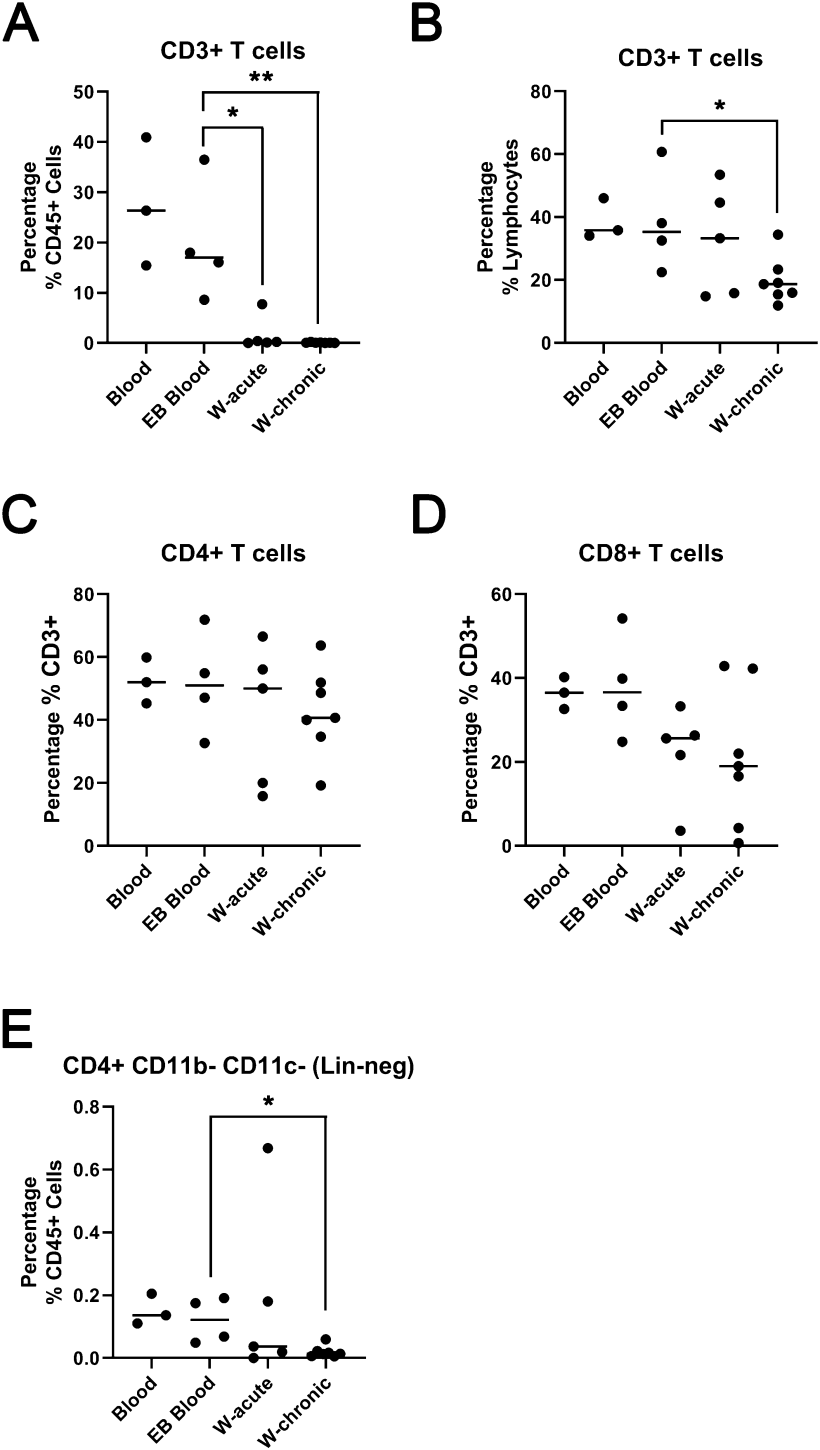
Subsets of T cells are readily identified in wound isolates. (**A**) Graph shows CD3 + cells as a percentage of CD45 + cells identified in healthy control blood (Blood, n = 3); EB blood (n = 4), acute wounds (W-acute, n = 5) and chronic wounds (W-chronic, n = 7). (**B**) Graph shows CD3+ cells as a percentage of gated lymphocytes. (**C**) Graph shows CD4+ cells as a percentage of CD3+ cells. (**D**) Graph shows CD4+ cells as a percentage of CD3+ cells. (**E**) Graph shows the population of CD45+ CD3− CD11c− CD16− CD4+ cells as a percentage of CD45+ cells. Bar shows median. *p < 0.05, **p < 0.01, Mann Whitney U test. Graphs made in GraphPad Prism 8, https://www.graphpad.com/scientific-software/prism/.

T lymphocytes, CD45+ CD3+ cells, were readily identified in wound isolations albeit significantly reduced compared with peripheral blood (Fig. 5A). The percentage of lymphocytes that were CD3+ was less in wounds compared with peripheral blood, significant so for chronic wounds (Fig. 5B). The proportion of CD4+ cells or CD8+ cells were not significantly different comparing peripheral blood and wounds, although the proportion of CD8+ cells were lower in chronic wounds (Fig. 5C,D). Interestingly, a CD45+ CD3− CD4+ lineage negative population of cells, which potentially mark a subset of innate lymphoid cells (ILC) known as lymphoid tissue inducer (LTi) cells^22^ were identified and significantly reduced in chronic wounds compared with peripheral blood (Fig. 5E).

### Molecular diagnosis using DNA extracted from wound dressings

As epidermolysis bullosa is an inherited condition, and clinical management is dependent on accurate identification of disease subtype, we assessed the utility of discarded dressings for molecular diagnosis. DNA isolated from cells recovered from wound dressings—either fresh or from proliferative adherent cells—successfully identified RDEB patient mutations with the use of massively parallel sequencing (Supplementary Fig. 4 and Supplementary Table 4).

## Discussion

In this study we describe a novel technique to directly sample viable cells present in wounds of patients with EB. Such an approach provides the opportunity to identify cellular composition within different categories of wounds and is non-invasive as sampling discarded dressings does not interfere with routine clinical care. In the case of EB the approach also offers an alternative to blood draw, skin biopsy, or buccal swab, for genomic DNA isolation and molecular diagnosis (Supplementary Fig. 4) and offers an alternate, non-invasive, option in challenging situations such as a neonate with extensive skin loss. Beyond DNA isolation, the ability to isolate adherent, proliferating cells from wound dressings provides a non-invasive method that has potential for a number of applications, particularly in EB where bone marrow transplantation^23^, mesenchymal stem cell infusions^24^ or autologous cell injections^19^ are in clinical use. In this instance the ability to identify cells at the site of wounds has the potential to indicate therapeutic efficacy. Furthermore, wound dressings offer a potential source for induced pluripotent stem cell technologies, an approach currently being developed for therapy in EB^25^.

Factors that influence the number of viable wound cells recovered from a discarded dressing in our study include wound size, the time a dressing is on a wound, and the time the dressing is stored prior to isolation (Fig. 2). These observations lead to a model where cells are continuously infiltrating wound dressings and remain viable for at least 48 h while removing a dressing and storing leads to a decrease in viability after 48 h. It should, however, be noted that two samples which were processed seven days after harvesting still retained > 10^7^ viable cells (Supplementary Table 1) which suggests that either certain populations are more durable and accumulate over time or certain populations may proliferate in the media used to store dressings prior to isolation (in “Materials and methods” section). Our flow cytometry data used wound samples harvested within 2 days and therefore we are unable to differentiate this question at this time. It is also important to note that our results could also be affected by mutliple other factors that potentially influence viability of wound cells recovered from discarded dressings. Such factors include, but are not limited to, the presence of emollients or the use of antibiotics or antiinflammatory drugs, either topically or systemically. Further, ongoing studies are addressing these and other variables with the goal of providing Clinical indicators of wound chronicity.

Interestingly, wound dressings from JEB patients yielded significantly more cells than those from RDEB patients (Fig. 2E). Surprisingly little work has directly compared wounds between these two groups of EB patients. A recent study in the hypomorphic mouse model of RDEB has identified a role for type VII collagen in lymphatic tissues^26^ and this could somehow lead to reduced cellular mobilization seen in RDEB patients compared with JEB patients. However, it is noted that laminin 332 also comprises the lymphatic tissue complex which type VII collagen supports^26^ and it is unknown whether similar defects are seen between RDEB and JEB patients at this time. Certainly our flow cytometry analysis did not include enough patients from each EB group to discern whether the observation of reduced cell number was driven by a particular sub-population of cells. With the exception of a single JEB patient with the lowest viability, no obvious differences in either percentage of CD45+ cells or proportion of granulocytes and monocytes between RDEB and JEB is seen, albeit in small numbers (Supplementary Table 3).

By directly comparing the viable cellular composition of acute and chronic wounds (some of which were from the same patient) we definitively show, in keeping with previous proteomic approaches, that persistent granulocytes are a prominent feature of wound chronicity (Fig. 3). We show that lymphocytes are reduced in wounds and that monocytes are reduced in chronic wounds when compared with peripheral blood (Fig. 3). These data support the hypothesis based on prior patient sampling and murine work that absent neutrophil clearance by macrophages delays wound healing^9,10,12,16^.

Neutrophils are one of the body’s first lines of immune defense, facilitated by their presence in large numbers in the circulation and their ability to rapidly accumulate at the site of wounds and infection. This rapid response is supported by neutrophil mobilization from bone marrow and also marginal pools present in different tissues. The central dogma of a short lived neutrophil with a single role in quick release of granule-stored effector molecules to combat microbial infection is changing as different sub-sets of neutrophils are being identified (reviewed in^27^). Whilst the goal of this study was not to stratify neutrophils in different wounds we did identify a shift in CD16 positivity (used to differentiate neutrophils from the granulocyte population) from blood to wounds (Fig. 4C,D). This is analogous to recent work identifying CD16^dim^ neutrophils with improved ability to contain *S. aureus* intracellularly^21^. Our own work in RDEB has identified dysbiosis in patient wounds with a significant presence of *S. aureus* in a European cohort^28^. The reduced CD16 expression may be identifying persistence of antibacterial neutrophils in EB patient wounds and future work comparing colonized with clean wounds in EB and non-EB patients will be informative in this context. Certainly, bacterial wound burden alters the local and systemic inflammatory cell response of the host and our approach offers the ability to study this influence by, for example, interrogating subsets of neutrophils present in different wounds from different patient groups.

Previous studies have identified differences from peripheral blood when comparing EB with healthy controls, showing reduced natural killer cell activity (not absolute numbers)^29^, as well as differences in monocyte and lymphocyte absolute numbers^30^. In this prior work the relative percentage of leukocyte subsets between EB and healthy controls were very similar while the study we present here shows potential increase of neutrophils and reduction of lymphocytes in EB peripheral blood compared with healthy controls, although these differences were not statistically significant (Fig. 3). These data are similar to observations comparing patients with diabetic foot ulcers which also identify increases in circulating neutrophils^20^, although the overall significance of these data have been challenged considering neutrophil numbers depend on a number of variables^31^. Further work will be needed to determine whether the number of leukocytes present in the circulation of EB patients does indeed differ from healthy controls or even genetically diagnosed sub types of the disease.

Finally, although we did not specifically look for “invariant NKT cells”, previously identified shown to promote wound healing in animal models^17^, we demonstrate the ability to identify rare subsets of lymphoid cells, such as LTi (Fig. 5E) confirming the utility of our approach for future comprehensive single cell studies of wound composition.

In conclusion we present a novel method for wound cell isolation that can identify differences between acute and chronic wounds, between JEB and RDEB patients, and provide a source of DNA for genetic diagnosis. Further development of this approach has the potential to delineate different subsets of innate and adaptive immune cells present in wounds which may predict chronicity. In addition, the approach may identify therapeutic targets and has potential to measure outcomes for treatments targeting the healing of chronic wounds.

## Materials and methods

### Study approval

Informed written consent was obtained from each patient prior sample collection. This study was performed in accordance with the Declaration of Helsinki and approved by all participating institutional review boards. Ethical approval was granted by the respective national Ethics committee [Austria: 415-E/2188/16-2019; Chile: Clínica Alemana Universidad del Desarrollo 2013-145; Stanford: #: 35473].

### Patient sampling

Patient recruitment has been performed at study centers in Austria (EB House Austria), Chile (DEBRA Chile), and Stanford, CA. Wounds were defined as acute if present for 21 days or less, and chronic if present more than 3 months. In total, we sampled 133 dressings from 51 patients, 40 diagnosed with RDEB, 10 diagnosed with JEB (9 *LAMB3*, 1 *COL17A1*) and one diagnosed with dominant dystrophic EB.

### Wound area calculations

During wound dressing sampling, a disposable paper ruler was placed next to the open wound and photographed. Pictures were processed with the software ImageJ by drawing the wound perimeter and calculating the area in square centimeters (cm^2^).

### Cell Isolation from wound dressings

Patient dressings were removed in the clinic as part of routine care but instead of discarding the used dressing the portion in direct contact with the wound was placed into a 50 ml falcon tube containing 40 ml of media as follows: (1) DMEM + 10% FBS + 2× Pen/Strep + 1× Fungizone or (2) RPMI 1640 + 10% FCS + 1% sodium-pyruvate + 1% l-glutamine + 1% Pen/Strep + 1% MEM non-essential amino acids + 0.0669 nM 2-mercaptoethanol + 1× Antibiotics/Antimycotics. A dressing coming from a single wound was used per 50 ml tube. Tubes were either stored at 4 °C overnight and processed the next day or shipped immediately to the laboratory at room temperature and processed upon arrival (up to 7 days after collection).

Cells from dressings were scraped off into PBS + Pen/Strep and collected in a 50 ml tube and combined with the remaining tissue and cells from the original media after collection with a centrifuge (300×*g* for 5 min at room temperature). At this point, 1/10 of the total cell/tissue volume was taken aside for adherent cell culture. 9/10 of the remaining volume was filtered through a 40 μm cell strainer (Corning®, Sigma) and pelleted by centrifugation for 5 min at 300×*g* at room temperature. Afterwards, cells were mixed and washed 3 times with 1× PBS + 1× A/A and total cell count was assessed with trypan blue staining and using a hemocytometer (Neubauer chamber).

### Adherent cell culture from dressings

1/10 of the total cell/tissue volume extracted from the dressing was centrifuged at 300×*g* for 5 min at room temperature. The pellet was resuspended in 5 ml of media (DMEM: Ham's F12 medium [3:1] supplemented with 10% FBS, 10 ng/ml of epidermal growth factor, 10^–10 ^M cholera toxin, 0.4 μg/ml of hydrocortisone, 5 μg/ml of transferrin, 5 μg/ml of insulin and 13 ng/ml liothyronine) + 1× Fungizone, 1× Pen/Strep, 1× Gentamicin, and plated on a T25 tissue culture flask. Adherent cells were cultured at 37 °C with 5% CO_2_.

### Leukocyte isolation from peripheral blood samples

Peripheral blood from four patients and three healthy donors were treated with ACK lysing buffer to remove red blood cells, following provider instructions. Briefly, 1 volume of EDTA blood was incubated with 20 volumes 1×ACK buffer for 5 min at room temperature and white cells were pellet by centrifugation at 300×*g* for 5 min. After one washing step with 1× PBS, cells were stained with trypan blue and viable cells were counted with a hemocytometer. Two million of viable cells were resuspended in FACS buffer (#420201 Biolegend) and subjected to antibody staining.

### Antibody staining and flow cytometry analyses of cells isolated from dressings

Cells isolated from wound dressings (only from the Chilean cohort and processed within 1 day from dressing collection) and peripheral blood leukocytes were used for flow cytometry analysis. Two million live cells were used for each condition, live/dead and all surface markers. Leukocytes and single color staining controls were used for setting up the populations and compensation, respectively. Cell viability (#L34971 Thermo Fisher) and Fc blockage (#422301 Biolegend) was performed prior surface marker staining. The antibodies used are detailed in Supplementary Table 2. Stained cells were either acquired immediately or fixed (2% Formaldehyde, 2% FBS in 1× PBS) and stored at 4C in darkness for up to 2 days prior acquisition. Data were acquired on a FACSAria (BD Biosciences) and analyzed with software FlowJo. For complete gating strategy, see Supplementary Fig. 2A.

### DNA extraction of cells isolated from dressings

Single cells freshly isolated from dressings were used for DNA extraction using the DNeasy Blood and Tissue Kit (QIAGEN), following provider instructions. The purity and integrity of isolated total DNA was confirmed by the A260/A280 ratio and 1% agarose gel electrophoresis, respectively.

### Mutational analysis

DNA samples were sent to the EB Haus Austria for Molecular diagnosis. Library was constructed by using an AmpliSeq EB-specific panel followed by performing NGS analyses on the Ion Torrent Personal Genome Machine platform. Variants were confirmed by Sanger sequencing.

### Statistical analysis

Statistical analysis was performed using Prism 8 (GraphPad Software, La Jolla, CA). For comparison of cell numbers and cell types the Mann–Whitney U test was used. Spearman Rank correlation was used to analyze potential relationships between sampling variables and viable cell number.

## Supplementary information

Supplementary Information

Supplementary Tables

## Data Availability

All data associated with this paper is included in the manuscript and the supplemental material.
