# Supplementary material for: Cells from discarded dressings differentiate chronic from acute wounds in patients with Epidermolysis Bullosa
Source: Sci Rep. 2020 Sep 15;10:15064. doi: 10.1038/s41598-020-71794-1 (PMC7492213; doi:10.1038/s41598-020-71794-1)

Supplementary Information

Cells from discarded dressings differentiate chronic from acute wounds in patients with Epidermolysis Bullosa

Ignacia Fuentes, Christina Guttmann-Gruber, Birgit Tockner, Anja Diem, Alfred Klausegger, Glenda Cofré-Araneda, Olga Figuera, Yessia Hidalgo PhD, Pilar Morandé, Francis Palisson, Boris Rebolledo-Jaramillo, María Joao Yubero, Raymond J Cho, Heather I Rishel, M. Peter Marinkovich, Joyce M.C. Teng, Timothy G Webster, Marco Prisco, Luis H. Eraso, Josefina Piñon Hofbauer, Andrew P. South.

Supplementary Tables 1-4 are available as a single, separate, Excel file.

Supplementary Table 1: Patient and dressing details.

Supplementary Table 2: Reagents for flow cytometry.

Supplementary Table 3: Flow cytometry results.

Supplementary Table 4: Genetic diagnosis.

Supplementary Figure 1: Cells isolated from Wound dressings. **A**. Phase contrast images of fresh isolations containing nucleated squamous cells (corneocytes): upper panel sample TJU2 and lower panel sample pEB212W1. **B**. Morphologies of different adherent cells isolated from wound dressings. **C**. Non-adherent cells comprise the majority of cells isolated from wound dressings: upper panel sample 201910231 on same day of isolation, lower panel shows non-adherent cells 12 days in culture after isolation, sample pEB220W2_1.

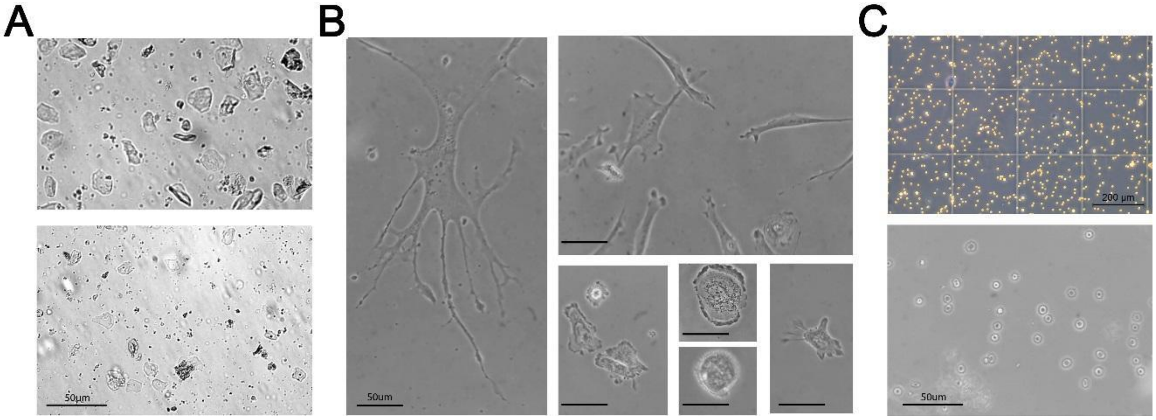

Supplementary Figure 2*: *Complete gating strategy and data processing used for immune cell profiling, giving rise to Supplementary Table 3 (A). **B**: Example of representative flow cytometry plot illustrating the differences in forward (x-axis) and side (y-axis) scatter from isolates of patient blood (pEB220) and matched wound dressing (pEB220W2_1). **C:** Example of representative flow cytometry plot illustrating the different populations detected in cells isolated from a patient blood (pEB220) and a wound dressing (pEB220W2_1). Gran = granulocytes; Mono = monocytes; Lymph = lymphocytes.

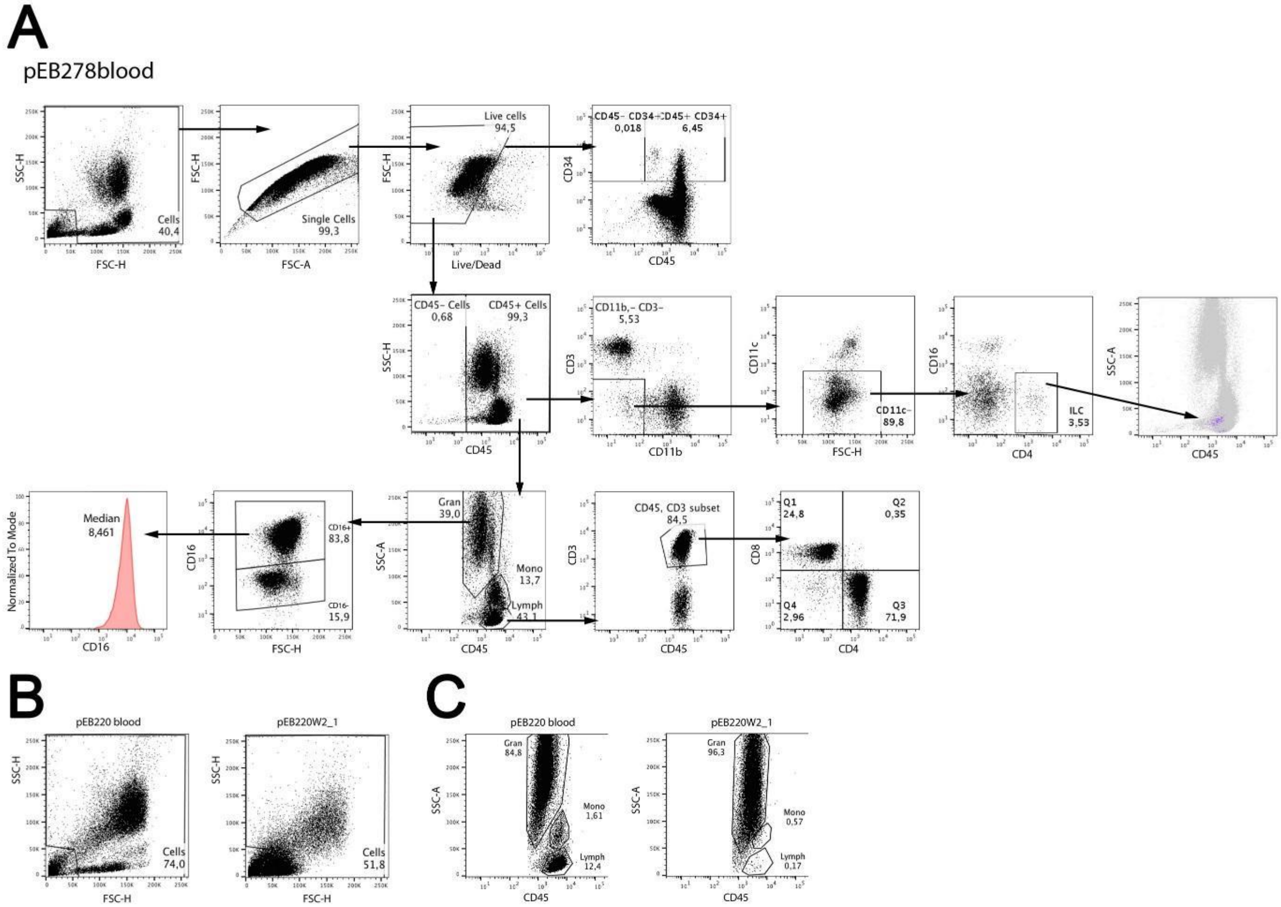

Supplementary Figure 3: Cell viability and CD45 positivity. **A**: Graph shows viable cells as a percentage of total cells identified in heathy control blood (blood, n=3); EB blood (n=4), acute wounds (W-acute, n=5) and chronic wounds (W-chronic, n=7). **B**: Graph shows CD45+ cells as a percentage of total viable (live) cells. Red bar shows median. * = p < 0.05, ** = p < 0.01, Mann Whitney U test.

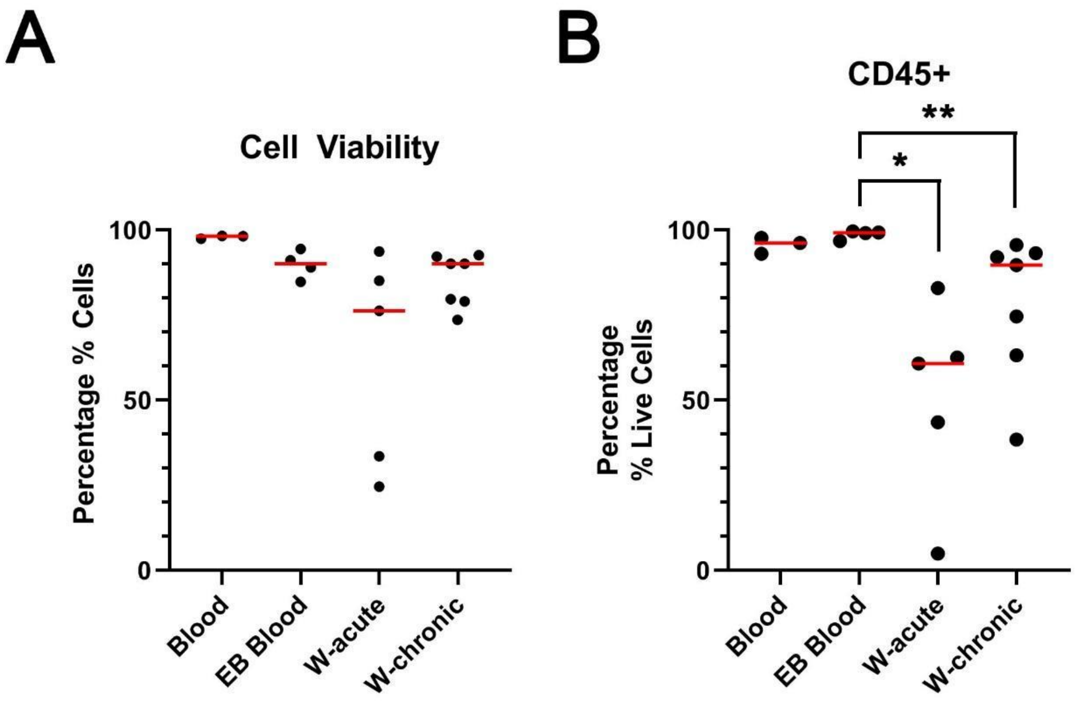

Supplementary Figure 4: Molecular diagnosis from wound dressings. **A**. Agarose gel showing good quality genomic DNA extracted from 2 EB patients processed 6 (pEB12W1_1) and 3 (pEB126W1_1) days post dressing collection. St = DNA size standard **B**. Sanger sequencing chromatograms confirming the homozygous mutation, c.6527dupC in exon 80 of *COL7A1* (extra C in blue), found initially in patient pEB12 by Next Generation Sequencing.

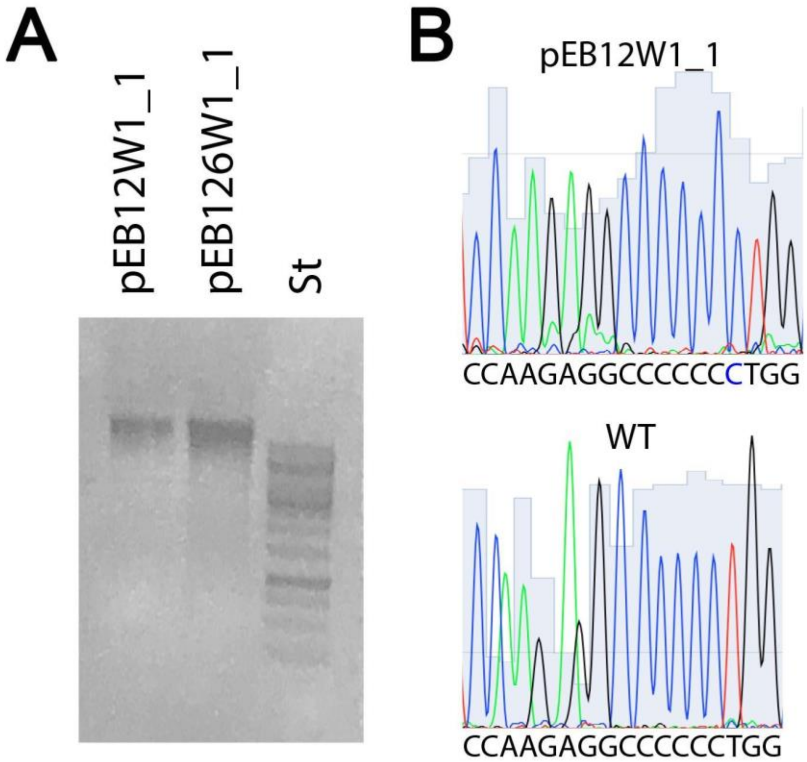

Supplement: Supplementary file 1 — Supplementary Information [file 41598_2020_71794_MOESM1_ESM.pdf]
